# How HEXACO Personality Traits Predict Different Selfie-Posting Behaviors among Adolescents and Young Adults

**DOI:** 10.3389/fpsyg.2016.02080

**Published:** 2017-01-10

**Authors:** Roberto Baiocco, Antonio Chirumbolo, Dora Bianchi, Salvatore Ioverno, Mara Morelli, Maria R. Nappa

**Affiliations:** ^1^Department of Social and Developmental Psychology, Sapienza University of RomeRome, Italy; ^2^Department of Dynamic and Clinical Psychology, Sapienza University of RomeRome, Italy

**Keywords:** selfies, HEXACO personality traits, adolescents, young adults, Honesy/Humility

## Abstract

Selfies are self-portrait photos shared on Social Networks. Previous literature has investigated how personality traits, and specifically narcissism, are associated with selfie-posting behaviors. In this contribution we investigated how selfie-posting behaviors are predicted by the six HEXACO personality traits, controlling for age, gender and sexual orientation. The Kinsey scale, three questions about the frequency of own selfies, group selfies and selfies with partner, and 60-item HEXACO Personality Inventory-Revised were administered to 750 young people from 13 to 30 years. Females, adolescents and not-exclusively heterosexual people posted more own selfies, and adolescents posted also more group selfies and selfies with partner. Moreover, lower Honesty/Humility, lower Conscientiousness, higher Emotionality and higher Extraversion significantly predict own selfies and group selfies. Finally, only lower Honesty/Humility and higher Emotionality predict selfies with partner. Theoretical and practical implications are provided.

## Introduction

A selfie is a self portrait photo typically taken with a Smartphone or a webcam and shared on social networks (Oxford Online Dictionaries, 2015). In the last years, selfies became very popular among young people all over the world. Recently, Sorokowski et al. (2015) suggested the distinction among three main kinds of selfies: selfies taken alone (or own selfies), selfies with a partner and group selfies, the latter also called groupies (Wang et al., in press). According to previous research, people usually take and post selfies for self-presentation, for seeking attention and feedback from peers (Kiprin, 2013; Katz and Crocker, 2015), for communicating, for archiving and for having entertainment (Sung et al., 2016).

Literature showed a relationship between online behaviors and different personality traits, founding that high extraversion, high openness to experience, high neuroticism and low conscientiousness predict more Social Network use (Ross et al., 2009; Ryan and Xenos, 2011; Eftekhar et al., 2014). Recently, studies started focusing also on the role of personality traits in selfie behaviors, employing the Five Factor Model as main personality theoretical framework, and found high extraversion and high neuroticism predicting more selfies (Qiu et al., 2015; Sorokowska et al., 2016).

In the last 10 years, the HEXACO model of personality structure enlightened interesting results that helped in understanding phenomena beyond the Five Factor Model (Lee and Ashton, 2004; Ashton and Lee, 2007; Ashton et al., 2014). In fact, HEXACO model is based on the same lexical and cross cultural studies from which originated the Five Factor model, but it is composed of six dimensions instead of five (Ashton et al., 2006): Honesty/Humility, Emotionality, Extraversion, Agreeableness, Conscientiousness, and Openness to experience.

The most important change is related to the introduction of a sixth factor, named Honesty/Humility. People with high Honesty/Humility are inclined to be sincere, fair, and unassuming. Conversely, people with low scores have a strong sense of self-importance and are inclined to flatter others and break the rules in order to get what they want. A recent review by Ashton et al. (2014) shed light on how Honesty/Humility dimension better predicts different variables compared to the Five Factor Model. These findings are consistent using both self-report and observer report methods. Moreover, low Honesty/Humility appears to be a good predictor of antisocial, manipulative and unethical behaviors, and to be strongly related to the Dark Triad dimensions (Ashton et al., 2014). Other differences between the Five Factor Model and the HEXACO model are related to Agreeableness and Emotionality factors (Ashton et al., 2004). In the HEXACO model, Emotionality describes a tendency to vulnerability, sentimentality and fearfulness vs. a tendency to fearlessness, detachment and toughness. Emotionality is similar to Neuroticism in the Big Five Factor Model, except for being less pejorative and for not describing individuals high on this dimension through ill-temper related terms. Agreeableness factor assesses a tendency to be cooperative, patient and lenient vs. a tendency to be ill-tempered, irritable and resentful. Thus, the Agreeableness in the HEXACO model is somewhat different from Agreeableness in the Big Five Factor Model since the latter excludes ill-temper related terms. The remaining factors, Extraversion, Conscientiousness and Openness to experience, are similar in both models. Specifically, Extraversion assesses the tendency to be optimist, secure in group interactions and confident in own social ability, vs. the feeling to be unpopular, unable and indifferent to social activity. High Conscientiousness describes people who tend to be organized, dependable, perfectionist, and obsessive, while low Conscientiousness describes people more flexible and spontaneous, but also negligent and unreliable. Finally, high levels in Openness to experience are related to curiosity, creativity, risk-taking and preference for novelty, while low levels are evident in preference for adhering to convention and predictable patterns.

Thus, the purpose of this study is to investigate the relationships between the HEXACO personality traits and three different kinds of selfie (own selfies, selfies with partner and group selfies), controlling for age, gender and sexual orientation, that in literature were found to be related with selfies and online behaviors (DeHaan et al., 2013; Chong et al., 2015; Jang et al., 2015; Dhir et al., 2016; Sorokowski et al., 2016). The HEXACO Model (Lee and Ashton, 2004), and specifically the Honesty/Humility dimension can help in deeply understanding these online behaviors. In fact, since high Honesty/Humility was found to be related to being fair and cooperative with others (Hilbig and Zettler, 2009; Chirumbolo and Leone, 2010), it is possible that people with low Honesty/Humility could post more selfies as a strategy to seek admiration and to take advantage from others to reach own social goals at the expense of communality on social networks.

Furthermore, previous research has shown that selfies-posting behaviors are predicted by high level of narcissism (Barry et al., 2015; Fox and Rooney, 2015; Sorokowski et al., 2015; Weiser, 2015; Halpern et al., 2016). In these studies narcissism is described as a multiform construct characterized by exhibitionism, vanity, grandiosity, exploitativeness, entitlement and desire of authority, aspects that could be motivated by the need for self-esteem regulation (Morf and Rhodewalt, 2001). Weiser (2015) underlined how both adaptive and maladaptive facets of narcissism can predict selfies posting. In fact, previous studies found that narcissism is strongly and negatively correlated with Honesty/Humility traits, and positively and strongly correlated with Extraversion traits (Lee and Ashton, 2005; Bresin and Gordon, 2011; Jonason and McCain, 2012). Thus these findings could support the link between honesty-humility and selfies, suggesting the utility of the HEXACO model in understanding selfies-posting behaviors. Moreover, Dark Triad traits (Paulhus and Williams, 2002), that include Narcissism, are well predicted by lower Honesty/Humility (Lee et al., 2013) and, thus it is plausible expecting that posting selfies could be more frequent among people with lower levels of Honesty/Humility.

Regarding demographics variables, studies on selfies found age and gender differences, showing that adolescents are more likely than young adults to post selfies on social network websites, and that girls usually share more selfies than boys (Jang et al., 2015; Dhir et al., 2016; Sorokowski et al., 2016). Conversely there is a lack in literature about sexual orientation differences in selfies. However, studies on online behaviors showed that sexual orientation seems to predict differences in the use of social networks: The Internet is commonly used by not heterosexual young people to find friends and romantic partners, in order to compensate the perceived difficulty of establishing offline contact with LGBT peers (DeHaan et al., 2013; Chong et al., 2015; Morelli et al., 2016).

In line with literature on online behaviors and personality traits (Ross et al., 2009; Ryan and Xenos, 2011; Eftekhar et al., 2014; Qiu et al., 2015; Sorokowska et al., 2016), and with studies on the relationship between selfies and narcissism (Barry et al., 2015; Fox and Rooney, 2015; Sorokowski et al., 2015; Weiser, 2015) that emerged being related to Honesty/Humility dimension of HEXACO model (Lee and Ashton, 2005), we hypothesized that low Honesty/Humility and high Extraversion could be related to all kinds of selfie-posting behaviors investigated. It is plausible that people with low Honesty/Humility could share more selfies in order to affirm their inflated beliefs about their positive self-view and extraverted people could share their selfies in order to improve their relationships and popularity.

Moreover, we expected that Conscientiousness would be negatively related to posting all kinds of selfies because people who are high in Conscientiousness are less involved in online behaviors and in the use of the Internet (Swickert et al., 2002; Butt and Phillips, 2008; Hughes et al., 2012), probably because they are engaged in offline activities and they considered Internet only a distraction from their tasks (Ross et al., 2009). Finally, we expected that high Emotionality could predict more selfie-posting behaviors because people high on this dimension may consider social networks as a safer place to express self-aspects compared to the off-line reality (Forest and Wood, 2012; Seidman, 2013), probably due to the association between Emotionality and social anxiety (Ashton et al., 2006).

## Materials and methods

### Participants

Participants were 750 adolescents and young adults (59.1% girls, *n* = 443) from 13 to 30 years old (*M*_age_ = 20.96; *SD*_age_ = 4.23), 82.1% (*n* = 616) reported to be exclusively heterosexual. Adolescents were recruited in secondary schools and an online survey was administered after obtaining written informed consents by parents and school authorities. Young adults were recruited via an online survey and they gave their informed consent by selecting “Yes, I accept to participate to this study” on the first page of the survey. This study was carried out in accordance with the recommendations of the Ethics Committee of the Department of Social and Developmental Psychology of Sapienza University of Rome, with written informed consent from all subjects. All subjects gave their informed consent in accordance with the Declaration of Helsinki. For minor participants, written informed consents were also obtained by parents and school authorities. The protocol was approved by the Ethics Committee of the Department of Developmental and Social Psychology of Sapienza University of Rome.

### Measures

#### Socio-demographic data

Participants were asked about socio-demographic information, such as gender, age.

#### Sexual orientation

Participants assessed their sexual orientation via the Kinsey Scale (Kinsey, 1948) on a 5-point Likert scale from 1 (exclusively heterosexual) to 5 (exclusively homosexual).

#### Selfie-posting behaviors

Selfies have been defined as self-portrait photos that people shared online (via social networks, Instagram, etc.). Three questions evaluated the frequency of different kinds of selfies, specifically own selfies, group selfies, and selfies with partner, during the last month on a 6-point Likert scale (from 1 = Never to 6 = More than once a day). A sample question was: “How often have you publicly posted your own selfies on social network during the last month?.” Only 454 participants (mean age = 21.95; standard deviation = 4.19; 63.7% girls, *n* = 289), who reported to currently be in a dating relationship (81.9% reported to be exclusively heterosexual, *n* = 372), completed questions about sharing selfies with their partner.

#### HEXACO personality traits

Personality traits were assessed with 60-item HEXACO Personality Inventory-Revised (Ashton and Lee, 2009). This inventory measured the six major dimensions of personality: Honesty/Humility, Emotionality, Extraversion, Agreeableness, Conscientiousness, and Openness to experience. Participants rated each item on a 5-point Likert scale (from1 = *Completely disagree* to 5 = *Completely agree*). Each scale showed a good reliability: Honesty/Humility (Cronbach alpha of.71), Emotionality (Cronbach alpha of.72), Extraversion (Cronbach alpha of.76), Agreeableness (Cronbach alpha of.74), Conscientiousness (Cronbach alpha of.71), and Openness to experience (Cronbach alpha of.70).

### Data analysis

First of all, we calculated correlations among all variables. Then, three hierarchical regression analyses were used in order to investigate which personality traits could predict the three kinds of selfies (i.e., own selfies, group selfies and selfies with partner), controlling for age, gender and sexual orientation. In the first step of each regression, socio-demographic variables, such as gender, age and sexual orientation were included as covariates. In the second step, the criterion was regressed on the six HEXACO personality traits.

## Results

First of all, we reported the zero-order correlations among the variables included in the study. Regarding correlations among different kinds of selfies and demographic variables, own selfies were negatively and weakly related to gender and age, and positively related to sexual orientation. Thus, own selfies were more frequently posted by females (66.1%) than males (55%), by adolescents (68.1%) than young adults (54.8%) and by not heterosexual (64.9%) than heterosexual participants (60.9%). Age was also negatively and modestly related to group selfies and selfies with partner: Group selfies were more reported by adolescents (81.5%) than young adults (66.8%) and also selfies with partner were more frequent among adolescents (75.8%) than young adults (57.1%). Regarding correlations between HEXACO personality traits and selfies, Honesty/Humility, Conscientiousness, and Openness to Experience were negatively and modestly related to all kind of selfies (i.e., own selfies, group selfies and selfies with partner). Moreover, own selfies were weakly and positively related to Emotionality and Extraversion, and negatively related to Agreeableness. Group selfies were weakly and positively related to Extraversion. Finally, correlations among HEXACO factors showed that Honesty/Humility was moderately and positively correlated with Emotionality, Agreeableness, Conscientiousness, and Openness to Experience. Emotionality was moderately and negatively correlated with Extraversion, that was found to be positively and moderately correlated with Conscientiousness and Openness to Experience. Finally, Conscientiousness was found to be positively and robustly correlated with Openness to Experience. Correlations among all the variables and descriptive statistics are reported in Table 1.

**Table 1:** **Correlations among variables**.

	**1**	**2**	**3**	**4**	**5**	**6**	**7**	**8**	**9**	**10**	**11**	**12**	**M**	**SD**
1. Gender	1												–	–
2. Age	−0.10^**^	1											20.96	4.23
3. Sexual Orientation	0.00	0.12^**^	1										–	–
4. H	−0.27^**^	0.13^**^	−0.02	1									3.48	0.68
5. E	−0.36^**^	0.01	0.01	0.16^**^	1								3.31	0.65
6. X	0.04	0.13^**^	−0.01	−0.01	−0.20^**^	1							3.25	0.67
7. A	0.01	0.03	0.01	0.26^**^	−0.01	−0.04	1						2.92	0.57
8. C	−0.13^**^	0.21^**^	−0.01	0.25^**^	0.08^*^	0.20^**^	0.08^*^	1					3.51	0.61
9. O	−0.10^**^	0.24^**^	0.20^**^	0.20^**^	0.07^*^	0.18^**^	0.04	0.31^**^	1				3.31	0.66
10. Own selfies^a^	−0.10^**^	−0.20^**^	0.08^*^	−0.15^**^	0.08^*^	0.08^*^	−0.10^**^	−0.17^**^	−0.10^**^	1			2.07	1.20
11. Group selfies^a^	0.01	−0.21^**^	0.01	−0.15^**^	0.01	0.15^**^	−0.05	−0.15^**^	−0.10^**^	0.61^**^	1		2.20	1.05
12. Selfies with partner^b^	−0.01	−0.20^**^	−0.01	−0.14^**^	0.06	0.01	−0.03	−0.11^*^	−0.14^**^	0.58^**^	0.53^**^	1	2.01	1.04

** p < 0.01;

* *p < 0.05*.

a *N = 750*.

b *n = 454*.

*Gender was coded as 0 = Females and 1 = Males. H, Honesty/Humility; E, Emotionality; X, Extraversion; A, Agreeableness; C, Conscientiousness; O, Openness to Experience*.

### Hexaco personality traits and own selfies

A hierarchical regression analysis was conducted following the previously described procedure to determine which personality traits predict sharing own selfies, controlling for gender, age, and sexual orientation. Gender, age and sexual orientation were entered in the first step as covariates. Altogether, they accounted for 6.5% of the variance, *R*^2^ = 0.065, *p* = 0.000. Both gender, β = 0.12, *p* = 0.001, age, β = −0.22, *p* = 0.000, and sexual orientation, β = 0.11, *p* = 0.002, emerged as significant predictors, with females (more than males), adolescents (more than young adults) and not-exclusively heterosexual people (more than exclusively heterosexual people) reporting to publicly share their own selfies on social networks. In the second step, in which HEXACO personality traits were added to the equation, 13.7% of the variance was accounted for, *R*^2^ = 0.137, with a significant increment of 7.2% in the explained variance, Δ*F*_(6, 740)_ = 10.26, *p* = 0.000. Gender, age and sexual orientation were still significant predictors but, controlling for these variables, lower Honesty/Humility, β = −0.12, *p* = 0.001, lower Conscientiousness, β = −0.14, *p* = 0.000, higher Emotionality, β = 0.10, *p* = 0.007, and higher Extraversion, β = 0.17, *p* = 0.000, turned out to be significant predictors of sharing own selfies. See Table 2 for regression coefficients.

**Table 2:** **Hierarchical regression analyses: own selfies, group selfies, and selfies with partner were, respectively, regressed on HEXACO personality traits, controlling for gender, age and sexual orientation**.

	**Selfies**					
	**Own selfies**		**Group selfies**		**Selfies with partner**	
**Predictor**	**Δ*R*^2^**	**β**	**Δ*R*^2^**	**β**	**Δ*R*^2^**	**β**
Step 1	0.06^***^		0.05^***^		0.04^***^	
Gender		0.12^***^		0.01		0.01
Age		−0.22^***^		−0.22^***^		−0.20^***^
Sexual Orientation		0.11^**^		0.04		0.01
Step 2	0.07^***^		0.07^***^		0.03^*^	
Gender		0.15^***^		0.04		0.02
Age		−0.19^***^		−0.20^***^		−0.16^**^
Sexual Orientation		0.12^**^		0.05		0.03
Honesty/humility		−0.12^**^		−0.11^**^		−0.11^*^
Emotionality		0.10^**^		0.07^*^		0.10^*^
Extraversion		0.17^***^		0.22^***^		0.07
Agreeableness		−0.05		0.004		0.01
Conscientiousness		−0.14^***^		−0.12^**^		−0.05
Openness to experience		−0.06		−0.05		−0.09
Total *R*^2^	0.13^***^		0.12^***^		0.07^*^	
*N*	750		750		454	

*Gender was coded as 0 = Males and 1 = Females*.

* p < 0.05;

** p < 0.01;

*** *p < 0.001*.

### Hexaco personality traits and group selfies

A hierarchical regression analysis was conducted following the previously described procedure to determine which personality traits predict sharing group selfies, controlling for gender, age and sexual orientation. Gender, age and sexual orientation were entered in the first step as covariates. Altogether, they accounted for 4.9% of the variance, *R*^2^ = 0.049, *p* = 0.000. Only age, β = −0.22, *p* = 0.000, emerged as a significant predictor, with adolescents (more than young adults) reporting to publicly share group selfies on social networks. In the second step, in which HEXACO personality traits were added to the equation, 11.8% of the variance was accounted for, *R*^2^ = 0.118, with a significant increment of 6.9% in the explained variance, Δ*F*_(6, 740)_ = 9.71, *p* = 0.000. Age was still a significant predictor but, controlling for these variables, lower Honesty/Humility, β = −0.11, *p* = 0.006, lower Conscientiousness, β = −0.12, *p* = 0.002, higher Emotionality, β = 0.07, *p* = 0.05, and higher Extraversion, β = 0.22, *p* = 0.000, turned out to be significant predictors of sharing group selfies. See Table 2 for regression coefficients.

### Hexaco personality traits and selfies with partner

A hierarchical regression analysis was conducted following the previously described procedure to determine which personality traits predict sharing selfies with partner, controlling for gender, age and sexual orientation. Gender, age and sexual orientation were entered in the first step as covariates. Altogether, they accounted for 4.1% of the variance, *R*^2^ = 0.041, *p* = 0.000. Only age, β = −0.20, *p* = 0.000, emerged as a significant predictor, with adolescents (more than young adults) reporting to share publicly on social networks selfies with partner. In the second step, in which HEXACO personality traits were added to the equation, 7.4% of the variance was accounted for, *R*^2^ = 0.074, with a significant increment of 3.3% in the explained variance, Δ*F*_(6, 444)_ = 2.65, *p* = 0.015. Age and sexual orientation were still significant predictors but, controlling for these variables, lower Honesty/Humility, β = −0.11, *p* = 0.026, and higher Emotionality, β = 0.10, *p* = 0.046, turned out to be significant predictors of sharing selfies with partner. See Table 2 for regression coefficients.

## Discussion

This study aimed to investigate the role of personality traits in the display of different typologies of selfies (i.e., own selfies, group selfies, and selfies with partner), taking into account gender, age and sexual orientation differences. We found that lower Honesty/Humility and higher Emotionality traits were associated with more own, group and romantic selfie-posting behaviors, while higher Extraversion and lower Conscientiousness traits were associated only with posting own and group selfies.

The present investigation is one of the first studies examining the role of the HEXACO model of personality in predicting selfie-taking behaviors. To the best of our knowledge, we could find only one unpublished exploratory paper (Paris and Pietschnig, 2015) reporting, in a merely descriptive manner, significant associations between HEXACO traits and travel selfie-taking behaviors. With respect to Paris and Pietschnig (2015), our study offered a more comprehensive view of the phenomenon by investigating different type of selfie behaviors in a more extensive sample, taking at the same time into account the impact of socio-demographic variables. Moreover, it is also worth to note that sexual orientation differences in selfie-posting behaviors have never been investigated in literature.

We consider the meaning and the implications related to the results of the present study, first regarding the associations between personality traits on different selfie-posting behaviors, and then regarding the role of the covariates effects (i.e., gender, age, sexual orientation) in such behaviors. Finally, we consider study limitations and possible directions for future research.

### Selfie-posting behaviors in the hexaco model of personality

Correlations between HEXACO personality traits and selfies-posting behaviors emerged in our study, although they were rather small. Nevertheless, large correlations were not expected, because selfie frequency at a given period could depend on many short-term situational factors, such as peer group norms, recency of having a phone, and current competing activities. Moreover, small correlations could be due to the fact that personality and selfies were assessed by self-report: Probably, higher correlations would be obtained from combination of self- and observer reports, as the aggregation across sources would likely increase the validity of the personality measures. In any case, regression analyses showed that some specific personality traits, according to the HEXACO model, were significantly associated with the three different selfie-posting behaviors: The effects of Honesty/Humility and Emotionality traits were consistent in the three typologies of selfies, while Extraversion and Conscientiousness traits seem to discriminate own selfies and group selfies from selfies with a partner.

Honesty/Humility was associated with a general low level of selfie-posting behaviors. People low on this personality dimension show high levels of slyness/deceit, pretentiousness and greed (Ashton et al., 2006). Since social networks allow most full control over self-presentation, we speculate that people low on Honesty/Humility dimension may tend more to select appealing photos of themselves in order to affirm their inflated beliefs about their positive self-view, especially of their physical appearance, social popularity, and status. It is also worth considering that Honesty/Humility has been found to strongly correlate with narcissistic aspects from other personality models (Lee and Ashton, 2005). Therefore, the negative association between Honesty/Humility and selfie-posting behaviors seems plausible and may indirectly support previous findings on the association of frequency of selfies with narcissism (Sorokowski et al., 2015; Sung et al., 2016). At the same time, we argue that the significant effect of the Honesty/Humility dimension moves beyond the well-known role of the narcissistic facets and gives a more nuanced understanding of the motivations that drive selfie-posting: Honesty/Humility is a trait level manifestation that is strictly related to social interactions, and translates into behaving fairly and cooperating with others in order to favor social equality (Chirumbolo and Leone, 2010). Thus, rather than for communication and interaction purposes, people low in Honesty/Humility may use selfies as self-regulatory strategies, such as admiration seeking and bragging and the selfie may indirectly represent a way to take advantage from others to reach own social goals at the expense of communality on social networks.

The positive association of Emotionality is also consistent with previous investigations on social media use (Seidman, 2013). People high in Emotionality tend to have large discrepancies between the actual and the ideal self and tend to present themselves differently from their self-perception. Since, as abovementioned, social networks allow people to control their self-presentations, selfies may represent an instrument to idealize selves online (Seidman, 2013). Since Emotionality is also associated to social anxiety (Ashton et al., 2006), other studies speculated that people high on this dimension may see the social networks as a safer place to express self-aspects compared to the off-line reality (Forest and Wood, 2012; Seidman, 2013). Thus, it is conceivable that people who show high Emotionality may tend more to use selfies as tools of expression and disclosure of hidden self-aspects not normally expressed in everyday life, because they probably have a greater need for emotional connection with others.

Consistent with our hypotheses, Extraversion was positively associated to own and group selfie-posting behaviors. This result is in line with findings that extraverted people more frequently engage in elaborate online self-presentations. High levels of extraversion are related to feeling positively about themselves and confident in groups, also in the online dimension. On the contrary, people with low levels of extraversion consider themselves unpopular and feel uncomfortable being at the center of attention, also in online contexts: Thus, they could post less frequently selfies. Specifically, according to previous studies (Marcus et al., 2006; Krämer and Winter, 2008; Sorokowska et al., 2016), extraverted people tend to present aspects of their own lives in a less restrained manner and to choose less conservative pictures of themselves compared to other people. Since, people who demonstrate high Extraversion are typically highly sociable (Ashton et al., 2006), posting selfies might also function as a display of willingness to seek out virtual social contact. If for people high in Emotionality is conceivable a “social compensation” hypothesis that proposes that social network may compensate for their weaker social skills, a “rich-get-richer” hypothesis may be applicable to extraverts who tend to gain more from social network usage as their offline sociability is transferred online (Correa et al., 2010). Indeed, since extraverted people are more active users of social network sites, it is likely that they are also more active in selfie-posting (Gosling et al., 2011). Contrary to another study where a significant correlation between Extraversion and partner selfies was reported (Sorokowska et al., 2016), we found no association between these two dimensions. Probably, after controlling for other personality and socio-demographic variables, the effect of Extraversion in our regression model may result less relevant for this specific kind of selfie-posting behavior. A possible explanation for this non-significant association is that taking romantic partner selfies may not be congruent with the social goals of extraverted people, namely the preference to seek social interaction (Costa and McCrae, 1992). Indeed, it is plausible that posting own and group selfies may stimulate social interactions to a greater extent compared to posting partner selfies that, conversely, might be driven mostly by other motivations.

Finally, the negative associations between Conscientiousness, own and group selfies suggest that more conscientious individuals are more likely to avoid showing personal aspects and more concerned about their privacy. This finding confirmed our hypothesis, according to which more conscientious people are less likely to spend a lot of their time on Social Networks because they consider them as a distraction from their tasks (Butt and Phillips, 2008). Moreover, in other studies Conscientiousness is related to authentic online self-presentation (Hall and Pennington, 2013) implying that conscientious people present themselves online in ways consistent with their self-perceptions. Based on this finding, it is speculated that selfie-posting behaviors are more likely in people who tend to show online self-presentations detached from the reality. The lack of association between Conscientiousness and selfie with partner may be interpreted taking into account the differences in the content of romantic photographs compared to own and group selfies: Romantic partner selfies tend to represent less inappropriate contents and are more consistent with social norms.

### Selfie-posting behaviors: the role of gender, age, and sexual orientation

Although not the focus of this paper, we observed some interesting results regarding the effect of the demographic covariates (gender, age and sexual orientation) on selfie-taking behaviors. Consistent with the literature (Jang et al., 2015; Dhir et al., 2016; Sorokowska et al., 2016), we found that girls participating in the study declared posting significantly more own selfies compared to boys. In order to explain these differences, prior findings suggested that women are more concerned compared to men with the creation of a positive picture of oneself online and the selection of pictures to show on social media (Haferkamp et al., 2012).

Moreover, it is conceivable that posting many pictures of oneself might be more socially acceptable among women compared to men and may reflect the nature of gender stereotypes that associate vanity and physically attractive self-presentation to women (Manago et al., 2008). Conversely, gender differences were not found with respect to posting romantic partner selfies and group selfies. The latter result is inconsistent with previous findings (Sorokowski et al., 2015, 2016) reporting that women publish a greater number of group selfies compared to men. Probably, such gender differences are less likely to be identified using a general measure of frequency of selfie-posting, as it was done in the present study, while it may be more detectable when accounting for the actual number of posted selfies (Sorokowski et al., 2015, 2016). Moreover, these inconsistent result could also be related to cultural differences that should be further investigated in cross-cultural studies.

In line with previous findings acknowledging that adolescents use social network sites and online communities more often than adults (Qiu et al., 2015; Dhir et al., 2016), in our study age was also negatively associated with all three typologies of selfie-posting behaviors.

Another interesting finding of the present study was the significant association between sexual orientation and selfie-posting behaviors. Specifically, our results showed that lesbian, gay and bisexual participants reported to post own selfies to a greater extent than the heterosexual counterparts. A first reasonable explanation for this result is strictly related to the abovementioned cultural views of taking selfies as a feminine behavior. It seems plausible to speculate that gay, lesbian, and bisexual people may feel less concerned to the gender stereotypes associated to taking selfies. A second hypothesis is mostly related to the usage of social network by gay, lesbian, and bisexual people. Indeed, dating apps have become popular particularly among not heterosexual people for practical reasons (DeHaan et al., 2013; Chong et al., 2015). Most of these apps employ global positioning system technology to facilitate connections with other users based on their current location and enable the users to see pictures from nearby users and chat with them (Grosskopf et al., 2014). The large usage of date apps that enable people to present themselves to multiple audiences simultaneously through a single selfie may affect people's self-presentation strategies.

### Limitation and future directions

The present study had a number of limitations that should be addressed in future research. Our findings were based on data from a self-reported survey. Ideally, future research could collect observable data of selfie-posting behaviors. However, the task of identifying appropriate measures for studies on online behaviors remains still very challenging since it requires time and special permissions that are difficult to obtain. Moreover, the observation of individuals' posts of selfies on social media is not exempt from self-selection bias in terms of one's willingness to participate in a study about selfie-posting behaviors.

Another limitation is that the study relied on cross-sectional data. It would be important to extend the present study with longitudinal data in order to assess the stability of the influence of personality traits and socio-demographic variables on selfie-posting behaviors over time.

Although the present study was interested in studying selfie-posting behaviors among adolescents, extending the study to older adults could be of considerable interest since according to the literature they may differ in their use of social networks (Pfeil et al., 2009). Finally, the categorization of the selfies (own, group and with romantic partner) may not be exhaustive: Future research should refine categories in a manner that potential significant information is not lose. For example, selfie can be further categorized based on their content: Some own selfies are focused on physical appearance, other are used to document special events and occasions. Mostly of the selfies might be coded based on contextual information accompanying them.

## Conclusion

In summary, although other studies have investigated specific correlates of personality in posting selfies (mostly, based on the evaluation of single trait effects), of note, the present study is the first to study the relationship between personality traits and different selfie-posting behaviors according to the HEXACO model. Additionally, the current study contributes to the existing literature on social media use by studying the relation between gender, age and sexual orientation, and posting selfies.

We found that, among personality traits, Honesty/Humility and Emotionality were the most consistent in predicting selfie-posting behaviors. Moreover, results showed that younger people, women and non-heterosexual people tend to take more own selfies compared to men and heterosexual people.

Despite the growing popularity and usage of posting selfies, yet the social, cognitive and psychological implications for posting selfie photographs to various online sites remain largely unknown. Our findings support the hypothesis that the motivations and the functions of this social phenomenon may be in part related to personality, gender, and sexual orientation based self-presentations. Moreover, this research may have important practical implications as selfie-posting behaviors represent a relevant aspect of many individuals' social interactions. Since selfie-posting behaviors are becoming more and more popular, the number and the type of selfies may provide additional important information on individual personality traits and self-representations. Specifically, this study suggests a key to understand online self-presentations in-depth. For example, as previously discussed, a selfie-posting behavior may express the need for affirming one's positive self-view, or for disclosing hidden self-aspects, or for seeking out virtual social contacts, that could be related to personality traits. Moreover, as social networking has become a widespread phenomenon in the lives of many people, examining personality traits may be helpful to differentiate normative vs. problematic behaviors in this domain. For instance, selfie-posting behaviors can have a potential negative impact on one's confidence and self-esteem for people with low Honesty/Humility, because selfies perpetuate the need for admiration and make them dependent on external feedback. Furthermore, people with high Emotionality may consider selfies an appealing venue for self-disclosure, thus selfie-posting behaviors could make them more vulnerable to undesirable responses as being rejected or ignored and perpetuate negative emotions. Of course, these are speculations and hypotheses to be tested in further studies that may provide new insights into how taking and posting selfies allow people to express, manage and develop their self-presentations and their social interactions.

## Author contributions

RB, MM, DB, AC, and MN originated the study. MM, DB, MN, and SI wrote the initial draft. MM and AC completed the analyses. RB and AC supervised the analyses. All the authors interpreted the findings and edited drafts of the article.

### Conflict of interest statement

The authors declare that the research was conducted in the absence of any commercial or financial relationships that could be construed as a potential conflict of interest.
